# Occupational Exposure to Ultrafine Particles in Metal Additive Manufacturing: A Qualitative and Quantitative Risk Assessment

**DOI:** 10.3390/ijerph18189788

**Published:** 2021-09-17

**Authors:** Marta Sousa, Pedro Arezes, Francisco Silva

**Affiliations:** 1 ALGORITMI Research Center, School of Engineering, University of Minho, 4800-058 Guimarães, Portugal; parezes@dps.uminho.pt (P.A.); fsilva@ctcv.pt (F.S.); 2 CATIM—Technological Center for the Metal Working Industry, 4100-414 Porto, Portugal; 3 CTCV—Technological Center for Ceramic and Glass, 3040-540 Coimbra, Portugal

**Keywords:** risk management, occupational exposure, incidental nanoparticles, control banding, ultrafine particles, exposure, metal additive manufacturing

## Abstract

Ultrafine particles (UFPs) can be released unintentionally during metal additive manufacturing (AM). Experts agree on the urgent need to increase the knowledge of the emerging risk of exposure to nanoparticles, although different points of view have arisen on how to do so. This article presents a case study conducted on a metal AM facility, focused on studying the exposure to incidental metallic UFP. It intends to serve as a pilot study on the application of different methodologies to manage this occupational risk, using qualitative and quantitative approaches that have been used to study exposure to engineered nanoparticles. Quantitative data were collected using a condensation particle counter (CPC), showing the maximum particle number concentration in manual cleaning tasks. Additionally, scanning electron microscopy (SEM) and energy dispersive X-ray analyzer (EDS) measurements were performed, showing no significant change in the particles’ chemical composition, size, or surface (rugosity) after printing. A qualitative approach was fulfilled using Control Banding Nanotool 2.0, which revealed different risk bands depending on the tasks performed. This article culminates in a critical analysis regarding the application of these two approaches in order to manage the occupational risk of exposure to incidental nanoparticles, raising the potential of combining both.

## 1. Introduction

Metal manufacturing processes have evolved significantly in the past couple of centuries. Nowadays, a metallic product can be manufactured using different technologies, such as casting, molding, forming, machining, and, more recently, additive manufacturing (AM), commonly known as 3D printing. AM is no longer exclusively a prototyping technology. It is now seen as a production process that is able to produce end-use parts for various applications, such as in the automotive industry, medicine, jewelry, and visual arts [1]. One of the advantages of metal 3D printing over more conventional manufacturing processes is the fact that it requires less material and less post-processing activities, which can lead to lower costs. On the other hand, one of the disadvantages is a lack of knowledge and consistent information on the occupational risks of 3D printing. Therefore, it is important to study the health implications of a variety of factors, including (but not limited to) exposure to raw materials and emissions, the safety criteria of 3D printing systems and machines, emissions toxicology, and best practices to control overall exposure [2].

Additionally, there is evidence that ultrafine particles (UFPs) are emitted during these processes, with different emission rates depending on the source materials, technology, modeling, and temperature used [3]. The UFPs’ nanometer scale allows them to reach and penetrate the lungs as well as bloodstream and internal organs [4]. Three different types of UFP can exist within workplaces: engineered nanoparticles (ENPs), incidental particles, and/or environmental background particles (natural and/or anthropogenic). Incidental nanoparticles are anthropogenic but are generated unintentionally and are usually physically and chemically heterogeneous compared to ENPs, which are manmade with very specific properties to suit a certain purpose [5]. There is now an increased concern centered on the consequent risks to and impact on human health when working with engineered nanomaterials. The number of studies recently published on this topic is proof of this concern [6]. However, there are workers exposed to incidental nanoparticles without research on the related risks.

Occupational incidental nanoparticles usually originated from industrial processes that require high temperature or massive energy [4], such as metal additive manufacturing, which uses, for example, electron beams and lasers as heat sources. Recent studies have been published on this topic, showing the importance of studying the occupational risk of exposure to UFPs in metal AM workstations [7,8,9]. Some metal-based nanoparticles can cause adverse effects at cellular and subcellular levels. Due to their size and characteristics, they can interact with DNA and proteins and are able to induce inflammatory responses and toxic effects in humans [10]. Therefore, increasing our knowledge on how to protect workers exposed to incidental nanoparticles in metalworking environments is crucial, especially considering the scarcity of standardized and systematic risk management methods for this purpose [3]. Consequently, pertinent questions arise and are yet to be answered: which approach should be used to manage risks related to incidental metal UFP exposure? Are current methodologies used to study the risk of exposure to ENMs sufficient and adequate for incidental ones?

The common approach to industrial hygiene is to define occupational exposure levels (OELs) for different coarse and fine fractions. However, currently, there are no regulations or limits for most types of incidental nanoparticle exposure.

Therefore, different approaches have been proposed and used to study, monitor, and control exposure to metal nanoparticles, although mostly for ENPs. In occupational contexts, it is common to use direct-reading instruments such as condensation particle counters (CPCs), optical particle counters (OPCs), electrical low-pressure impactors (ELPIs), and/or scanning mobility particle sizers (SMPSs). Other strategies use filter-based samples and later analyze the collected material via, for example, scanning electron microscopy (SEM), transmission electron microscopy (TEM)s and/or energy-dispersive X-ray analyzers (EDSs), which provide a structural and chemical analysis [11,12,13,14,15,16].

However, former experience in chemical safety assessments and industrial hygiene shows that quantification is not enough to protect workers and avoid negative health impacts. It is necessary to establishing reference levels, such as OELs or derived no-effect levels (DNELs), which create a connection between risk assessment and control measures. These limits have been difficult to establish due to a lack of information on particle toxicology, metrics considerations, the high diversity of particles, and uncertainties about their hazardous properties [17]. Therefore, qualitative approaches to assessing the risk of exposure to nanoparticles should provide an alternative or complementary addition to quantitative analysis [18].

This article aims to investigate potential exposure to incidental ultrafine particles during metal AM through a case study conducted in an industrial workplace using laser cladding technology. Additionally, this study will serve as a pilot study to explore the suitability of combining both quantitative and qualitative approaches to manage this occupational risk.

## 2. Materials and Methods

### 2.1. Operation Conditions and Materials

**Figure 1:**
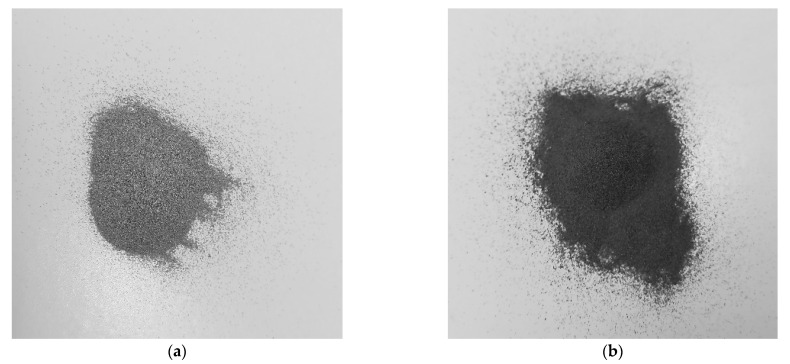
Photograph of inert gas-atomized powders used for laser cladding applications: (**a**) cobalt–chromium–silicon–carbon alloy (Powder 1); (**b**) tungsten carbide–nickel alloy (Powder 2).

Data for this study were collected in a company specialized in technical coatings for industrial applications using laser cladding. This equipment can use different inert gas-atomized powders, specifically designed for laser cladding applications. Therefore, two raw materials were considered: a cobalt–chromium–silicon–carbon alloy (Powder 1) and a tungsten carbide–nickel alloy (Powder 2) (Figure 1).

Data gathering included a sample of each raw powder, the technical and material safety data sheets of the powders, as well as details on the operation conditions for each material and on-site measurements.

### 2.2. Quantitative Assessment

The following equipment was used for on-site measurements:

- A condensation particle counter (CPC), TSI^®®^ Model 3007, to measure particle number concentration, with a particle size range of 10 nm to >1 µm in 1 s time resolution;
- A thermo-hygrometer, TSI^®®^ Model 9545, to measure air velocity, room temperature, and relative humidity;
- A personal air sampling pump (SKC AirChek^®®^ TOUCH) to collect samples for subsequent scanning electron microscopy (SEM) and energy-dispersive X-ray spectroscopy (EDS) analysis. The samples were collected using mixed cellulose ester (MCE) membrane filters (0.8 μm pore) that met NIOSH specifications for analysis of airborne metals. Additionally, these filters can collect particles with high efficiency, including particles much smaller than their nominal pore size [19].

Initially, background measurements were performed before any printing activity and with the machine still turned off. Later, two trials were performed: Trial 1 during laser cladding with a cobalt–chromium–silicon–carbon alloy as the raw material (Powder 1); Trial 2, while using a tungsten carbide–nickel alloy alloy (Powder 2) (Figure 1). Each trial consisted of three measurements during three different tasks, during which the worker is considered to be more exposed to AM emissions. These tasks are listed in Table 1.

It is important to highlight the fact that measurements during the additive manufacturing process itself (that is, with the machine working) were not performed since the machine works fully closed and has an incorporated exhausting system working during printing activity. While the machine is working, the worker stands outside the chamber, near the control panel, thus significantly reducing exposure.

### 2.3. Qualitative Assessment

Regarding qualitative approaches, control banding methodology has been used to study exposure to nanoparticles, mostly ENPs. In 2016, it was highlighted as the approach that can deliver better endorsement for occupational analysis in this field [20]. Among different control banding models, Control Banding Nanotool (version 2.0) was the one chosen for this case study since it shows the potential to be used to study occupational exposure to incidental nanoparticles [21].

The pilot CB Nanotool was created in 2008 by Paik et al. [22] and adapted one year later by Zalk et al. [23]. In 2019, the authors validated this CB model [24]. This method was designed to assess the risk of exposure to engineered nanomaterials. Regardless, this method has been previously applied to incidental nanoparticles [25].

**Figure 2:**
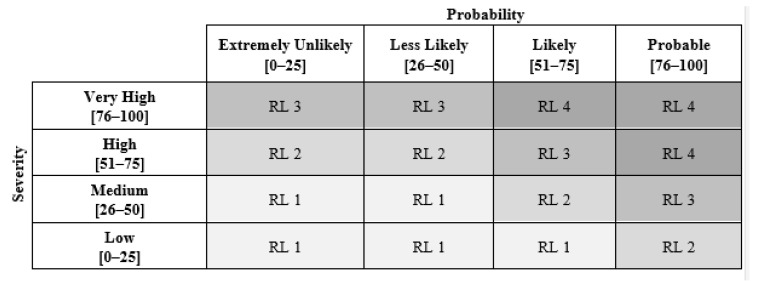
CB Nanotool risk level matrix, adapted from Zalk et al. [23].

CB Nanotool 2.0 assigns a severity score and a probability score to a particular operation, allowing the determination of a risk level using a four-by-four matrix (Figure 2).

In Figure 2, RL stands for risk level, and each of the four risk levels is related to a control band: RL 1 corresponds to general ventilation, RL 2 to fume hoods or local exhaust ventilation, RL 3 to containment, and RL 4 to seeking specialist advice.

The severity score is dependent on factors related to the nanomaterial (70% of the severity score) and the parent material (30% of the severity score). Nanomaterial (NM) factors include:

- surface chemistry (points: high = 10; medium = 5; low = 0; unknown = 7.5);
- particle shape (points: tubular/fibrous = 10; anisotropic = 5; compact/spherical = 0; unknown = 7.5);
- particle diameter (points: 1–10 nm = 10; 11–40 nm = 5; >40 nm = 0; unknown = 7.5);
- solubility (points: insoluble = 10; soluble = 5; unknown = 7.5);
- carcinogenicity (points: yes = 6; no = 0; unknown = 4.5);
- reproductive toxicity (points: yes = 6; no = 0; unknown = 4.5);
- mutagenicity (points: yes = 6; no = 0; unknown = 4.5);
- dermal toxicity (points: yes = 6; no = 0; unknown = 4.5);
- asthmagen (points: yes = 6; no = 0; unknown = 4.5).

On the other hand, parent material (PM) factors are scored by considering:

- occupational exposure limits (OELs) (points: <10 µg/m^3^ = 10; 10–100 µg/m^3^ = 5; 101–1000 µg/m^3^ = 2.5; unknown = 7.5; >1000 µg/m^3^ = 0);
- carcinogenicity (points: yes = 4; no = 0; unknown = 3);
- reproductive toxicity (points: yes = 4; no = 0; unknown = 3);
- mutagenicity (points: yes = 4; no = 0; unknown = 3);
- dermal toxicity (points: yes = 4; no = 0; unknown = 3);
- asthmagen (points: yes = 4; no = 0; unknown = 3).

The probability score considers factors related to the workers’ exposure to nanomaterials:

- estimated amount of material used (points: >100 mg = 25; 11–100 mg = 12.5; 0–10 mg = 6.25; unknown = 18.75);
- dustiness/mistiness (points: high = 30; medium = 15; low = 7.5; unknown = 22.5);
- number of employees with similar exposure (points: >15 = 15; 11–15 = 10; 6–10 = 5; 1–5 = 0; unknown = 11.25);
- frequency of operation (points: daily = 15; weekly = 10; monthly = 5; >monthly = 0; unknown =11.25);
- duration of operation (points: >4 h = 15; 1–4 h = 10; 30–60 min = 5; <30 min = 0; unknown = 11.25).

## 3. Results

### 3.1. Quantitative Assessment Results

#### 3.1.1. On-Site Measurements Results

Temperature, relative humidity, and air velocity were measured to characterize the environmental conditions of the workplace under study and to give insight into these conditions for follow-up experiments. The results are presented in Table 2.

**Figure 3:**
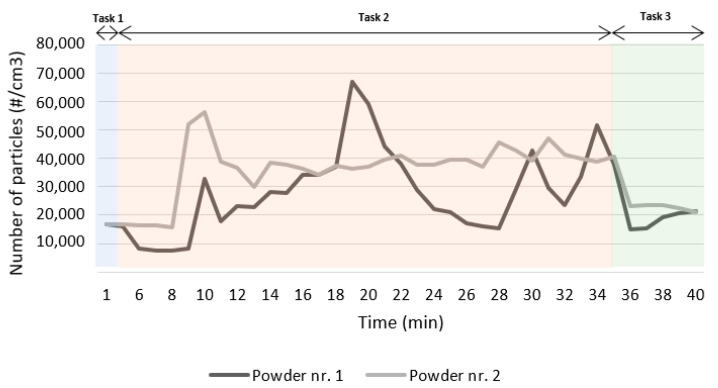
Number particle concentration (#/cm^3^) measured over the operation period with the CPC during both trials.

The CPC allowed the measurement of particle number concentration before any task was performed (background measure) and during each one of the three tasks considered likely to expose workers to metal UFPs for each trial (as described in Table 1). The corresponding results are presented in Table 3. Additionally, in Figure 3, it is possible to verify the evolution of the concentration of airborne particles over time for the three tasks under study and for each one of the trials performed.

#### 3.1.2. SEM and EDS Results

**Figure 4:**
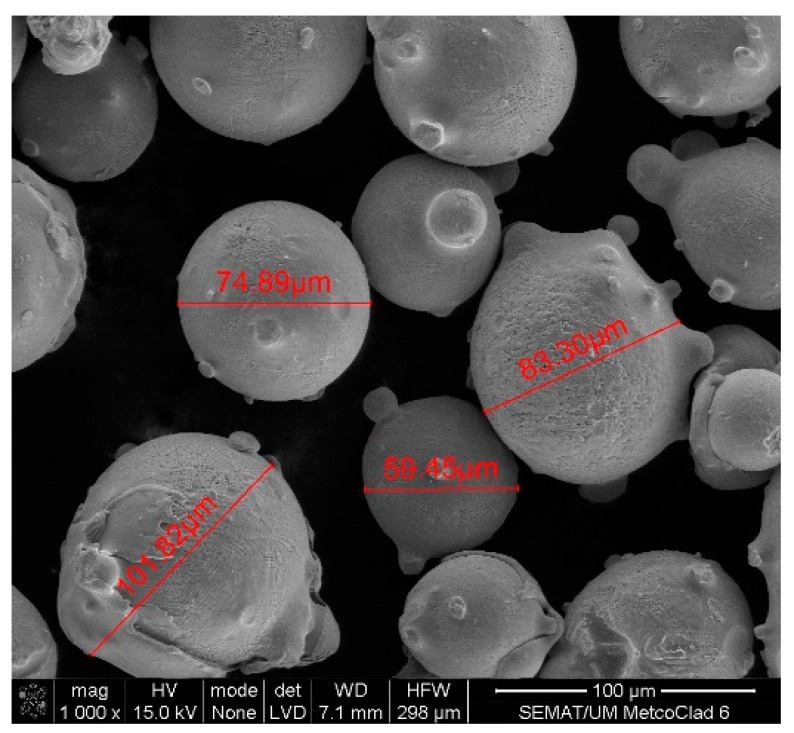
SEM analysis results: Raw Powder 1 sample—cobalt–chromium–silicon–carbon alloy.

**Figure 5:**
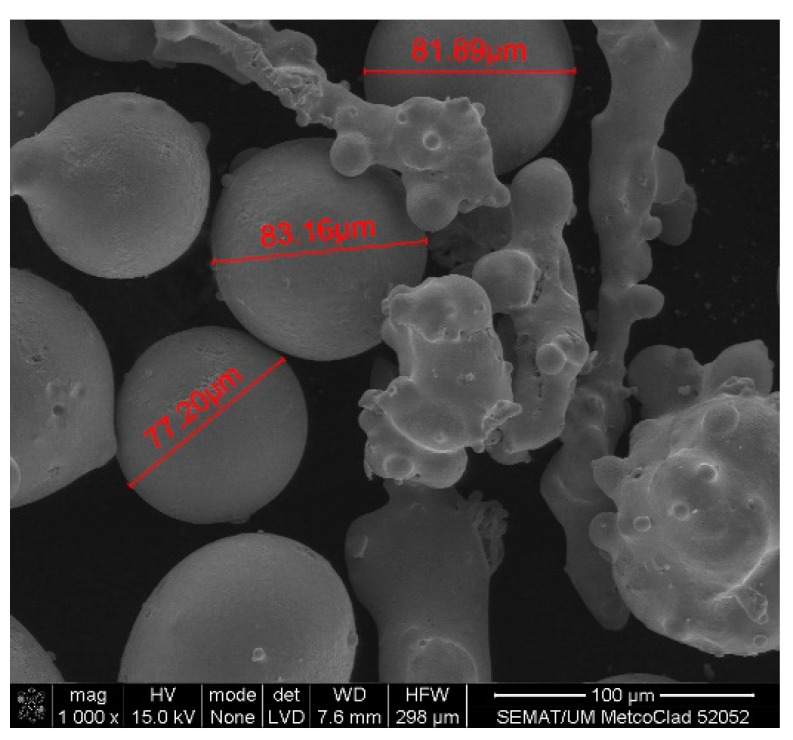
SEM analysis results: Raw Powder 2 sample—tungsten carbide–nickel alloy.

**Figure 6:**
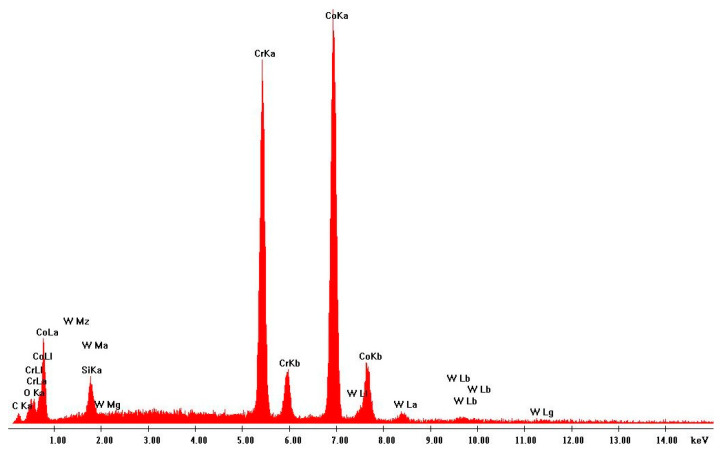
EDS analysis results: Raw Powder 1 (cobalt–chromium–silicon–carbon alloy).

**Figure 7:**
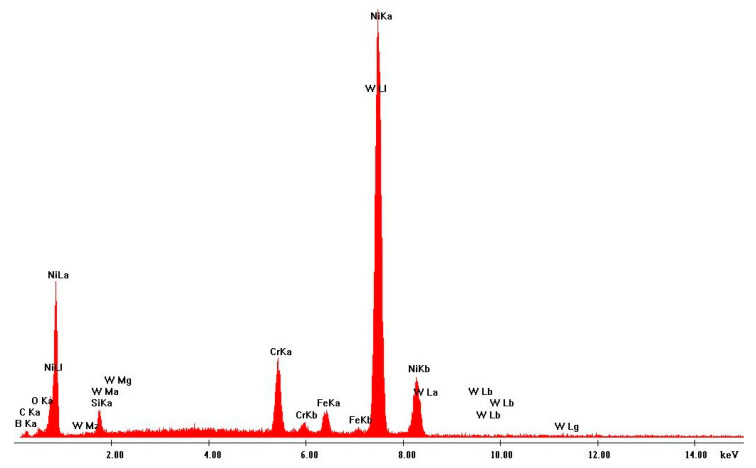
EDS analysis results: Raw Powder 2 (tungsten carbide–nickel alloy).

**Figure 8:**
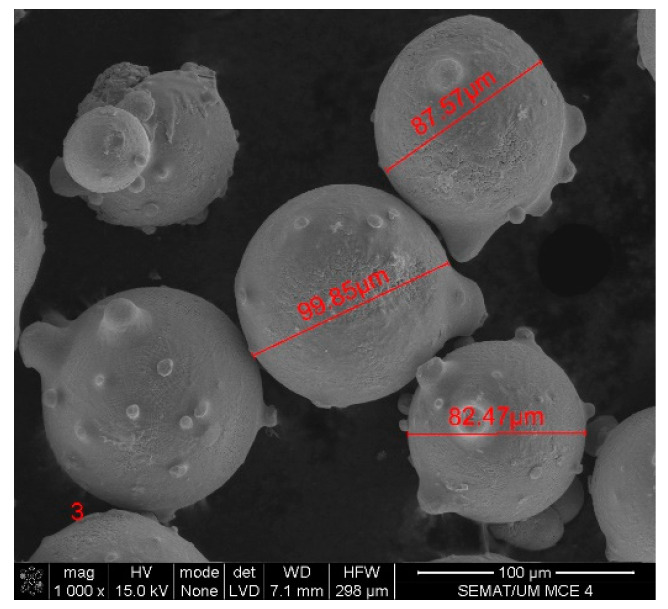
SEM analysis results: Sample collected during Trial 1 with the cobalt–chromium–silicon–carbon alloy.

**Figure 9:**
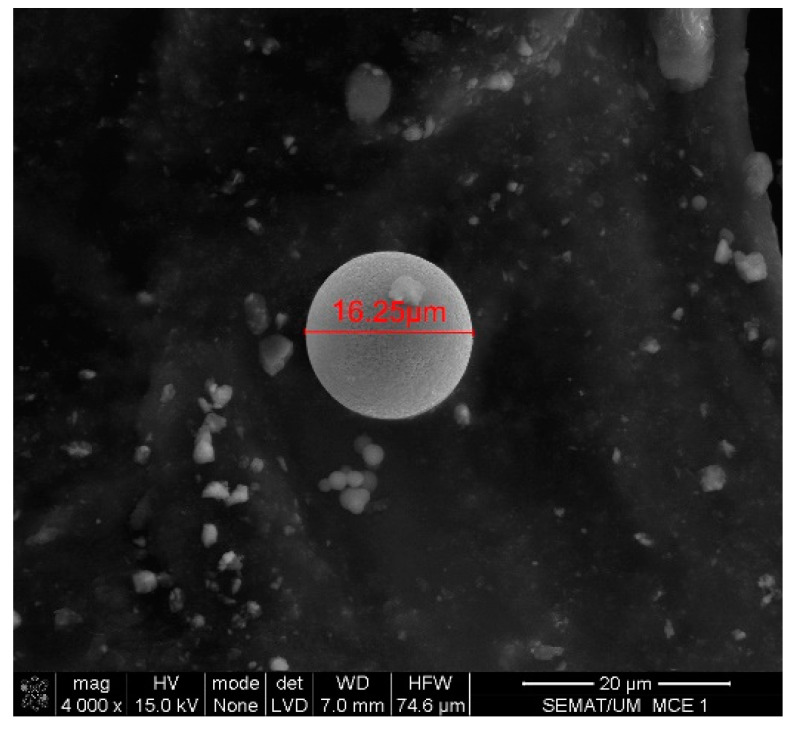
SEM analysis results: Sample collected during Trial 2 with the tungsten carbide–nickel alloy.

**Figure 10:**
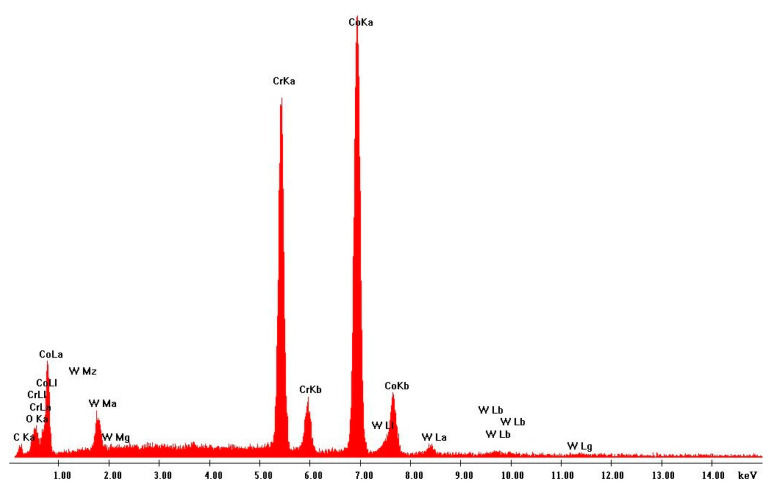
EDS analysis: sample collected during Trial 1 with the cobalt–chromium–silicon–carbon alloy.

**Figure 11:**
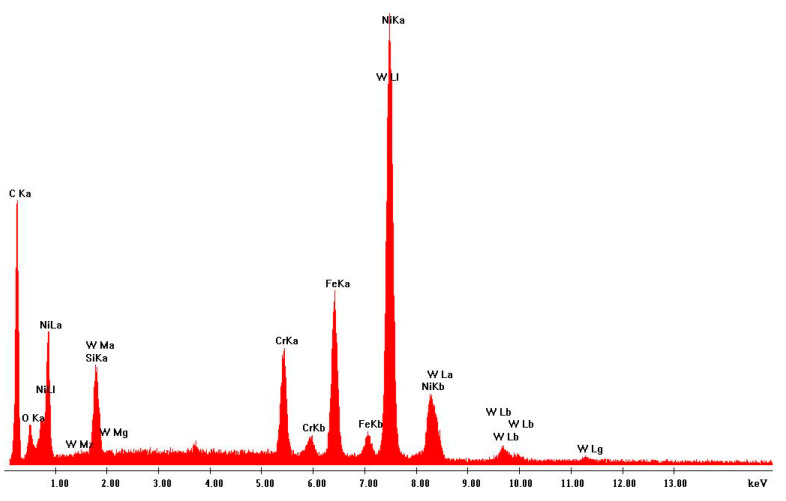
EDS analysis: sample collected during Trial 2 with the tungsten carbide–nickel alloy.

Scanning electron microscopy (SEM) was performed on the collected samples to increase data on the size and shape characterization of the raw materials and particles released into the work environment. Additionally, energy-dispersive X-ray spectroscopy (EDS) analysis was carried out to verify the elementary composition of both raw materials and consequent emissions after laser cladding. SEM and EDS analysis results of the raw powders (before laser operation) are presented in Figure 4, Figure 5, Figure 6 and Figure 7. Figure 8, Figure 9, Figure 10 and Figure 11 show the SEM and EDS results of the individual samples collected during the two trials.

### 3.2. Qualitative Assessment Results

CB Nanotool 2.0 was used to assess the risk of exposure to incidental nanoparticles in both trials. Table 4 shows the results of the application of this method for laser cladding with Powder 1 as the parent material (PM). On the other hand, Table 5 shows the results considering Alloy Nr. 2 as the PM.

## 4. Discussion

### 4.1. Quantitative Assessment

Considering that metal AM processes have the potential of emitting UFPs, the lowest mean number particle concentration was expected on background measurements. This condition was verified for both trials, as shown in Table 3.

Observing the same table, it is possible to confirm that both trials produced similar results. In both cases, the highest mean particle number concentration was obtained during Task 2. Figure 3 shows that the highest value was measured during this task, inside the machine operating area, while the worker removes and cleans the metal part. For Task 2, the mean value measured exceeds more than twice the background levels, being higher when printing with the cobalt–chromium–silicon–carbon alloy (presenting a 181% increase).

The lowest mean particle number concentration, after background, was obtained while pouring the raw powder into the machine container (this task is performed outside the machine’s operating area). Using the data of Table 3, it is possible to infer that the CPC results revealed around a 25% increase in concentration compared to background levels during Task 1 in both trials; during Task 3, there was an increase of 37% on Trial 1 and 70% on Trial 2.

The CPC results showed consistency, given that the lowest and highest values of mean particle number concentration were obtained for the same tasks, independently of which powder was being used (as emphasized in Figure 3). These results suggest greater exposure to particles while the worker is inside the machine operating area, after the AM process occurs.

EDS analysis for both samples of raw powder corroborates the information of the technical data sheet of each material on the materials’ chemical elemental composition (Figure 6 and Figure 7). The composition of Powder 1 is mainly cobalt and chromium, although other metals are naturally present in the alloy. The main elements of Powder 2 are tungsten and nickel. Figure 10 and Figure 11 show the EDS analysis of the samples collected during the AM process in the environment, showing that the chemical composition of the particles emitted is identical to their raw material. However, for Powder 2, there are some subtle differences that may indicate oxidation.

SEM detected medium-size particles (ranged from 1 to 100 μm), as shown in Figure 4 and Figure 7. These images reveal that the particles did not show any visible alteration to their size or surface (rugosity) after laser action.

After analyzing all the quantitative results obtained in this case study, it is not possible to clearly assess the risk of exposure to UFPs as there is no occupational limit value for incidental nanoparticles to function as a reference. Even after the processing of the quantitative data collected, the results are not sufficient to say with certainty that workers are exposed (or not) to UFP concentration levels that may have an impact on their health and safety conditions. Without OELs, or at least more reference levels, the results also lack information on whether the workstation under analysis requires the implementation of additional control measures to protect workers from the risk of exposure to UFPs.

### 4.2. Qualitative Assessment

The criteria considered during the application of CB Nanotool 2.0 on Trial 1 are present in Table 4. As the main components of the alloy are cobalt and chromium, the cobalt compounds’ OEL was considered as parent material OEL since this metal has the lowest OEL (most penalizing). Nevertheless, cumulative effects may be the worst-case scenario. Parent material carcinogenicity, reproductive, mutagenicity, dermal toxicity, and asthmagen factors were rated based on the classification of this product according to CLP Regulations Repr. 2 (H361f), Skin Sens. 1 (H317), and Resp. Sens. 1 (H334).

Nanomaterial factors were classified as “unknown” since there was no evidence of these characteristics for the incidental nanoparticles released. Different analysis and equipment would be necessary to classify the incidental nanoparticles, considering the surface chemistry, solubility, carcinogenicity, and other characteristics questioned in this method. Assuming these nine criteria as “unknown” and to make no assumptions, the score of the severity band was the same for the three tasks: 72 points. On the other hand, regarding probability, different scores were obtained for each task, as exposure time was different in each task.

The same line of thought was considered in Trial 2, with the tungsten and nickel alloy. The results are presented in Table 5. Nickel compounds have the lowest OEL, so, for that reason, it was considered as the PM OEL. Since this metal powder is classified as Carc. 2 (H351) and Skin Sens. 1 (H317) according to CLP Regulation classification (material safety data sheet data), PM carcinogenicity and dermal toxicity were considered applicable. Similar to Trial 1, no information on incidental nanoparticles was available to score the NM factors other than “unknown”. Therefore, the severity score was 63 for all tasks, 9 points lower compared to Trial 1. Concerning the probability score, as exposure time was higher for Task 2 and lower for Task 1, different scores were obtained for the three tasks.

After using CB Nanotool 2.0 for both case studies, a Risk Level 4—seek specialist advice was obtained for Task 2 in both trials. For Tasks 1 and 3, regardless of the raw powder used, the risk level band obtained was 3, meaning containment is the recommended control measure to reduce the risk of exposure to nanomaterials.

Contrary to the quantitative data, one of the outcomes of this approach is a tangible risk level that allows the user to conclude on the complementary control measures needed, even if it is based on some assumptions.

### 4.3. Comparision between Qualitative and Quantitative Assessments

The results of the quantitative and qualitative analyses are consistent in this pilot study since both approaches underscore Task 2 in relation to others, especially in relation to Task 1.

The qualitative approach used in this case study leads to two important results: a quantifiable risk level and specific control measures to prevent worker exposure. The control banding approach allows the user to understand which step to take towards reducing and preventing the risk of exposure based on the risk level obtained.

As mentioned before, CB Nanotool 2.0 was designed for engineered nanomaterials and although it allows the classification of “unknown” in certain parameters, there is a level of uncertainty introduced by these assumptions for incidental nanoparticles. These hypotheses may overestimate the risk. Nonetheless, with adjustments and more background data on the incidental nanoparticles under study, this tool has the potential to be eligible to assess the risk of exposure to incidental nanoparticles.

On the other hand, a quantitative approach offers less biased data and information, which may be very useful for decision-making. The results show higher mean particle number concentration when the worker is inside the machine and lower mean particle number concentration during background measurements, precisely as expected. Thus, the results suggest reliable measurements; they lack information on exposure to UFPs and, moreover, a clear understanding of the occupational risk of exposure. There are no established OEL, reference values, or similar guidelines for incidental nanoparticles, which makes it difficult to interpret the results and, consequently, define adequate control measures to reduce the risk of exposure to UFPs.

However, this research may corroborate the potential of using both approaches in combination. Quantitative results appear to be more accurate and less biased, not being dependent on the user’s interpretation. Thus, this approach, considering the information available nowadays on incidental nanoparticles, may lead to doubts on the meaning and interpretation of the values obtained. Still, these results may be good input for a more accurate qualitative approach, which is built on many assumptions. For incidental nanoparticles, there is not much background information, so any available data on UFPs, for example, on concentration, chemical composition, shape, and size, can be valuable.

## 5. Conclusions

The main objective of this case study was to investigate the potential exposure to incidental nanoparticles during metal AM and to be a pilot study on the suitability of both quantitative and qualitative approaches to managing this occupational risk.

The results of the quantitative analysis revealed less bias, although it also highlighted a lack of occupational limits for comparison. This is a significant limitation when using a quantitative approach to study incidental nanoparticles. Additionally, the quantitative approach does not give insights on how to control the risk of exposure to UFPs. In this case study, this insight was given by the qualitative method used (CB Nanotool 2.0). However, this method was not designed to manage risks related to incidental nanoparticles, so there is some uncertainty associated with the analysis. The biggest difficulty in using this approach for incidental nanoparticles is the lack of background information on the particles (such as size, shape, and solubility, among others). Therefore, one of the most significant findings of this case study is that qualitative methods used to assess the risk of exposure to incidental nanoparticles should have different inputs other than the ones designed for ENPs. If not, more qualitative data are needed for incidental nanoparticles.

In conclusion, it is possible to realize that there is an opportunity when using these approaches in a combined manner: on one hand, the qualitative assessment gives inputs on control measures, and, on the other hand, the quantitative approach provides more detailed information about UFPs that may provide more accurate inputs for the qualitative methodology used. This pilot study may give a good insight for future research to explore the potential of combining these two approaches to create solutions to manage the risk of exposure to incidental NMs.

## Figures and Tables

**Table 1:** Description of the tasks under study.

Task 1	Manual handling of the powder to fill the machine container with raw powder
Task 2	Removing and cleaning the final part from the machine after the coating process is completed (inside the machine operating area)
Task 3	Removing the remains of the powder and cleaning the powder container

**Table 2:** Results of the measurements performed with the thermo-hygrometer: air velocity, room temperature, and relative humidity.

Assessed Parameters	Background (Before Coating Operations)	Near the Machine Powder Tank	Inside the Chamber (Machine)
Temperature [°C]	22.5	23.1	22.6
Relative Humidity [%]	44.7	45.2	45.0
Air Velocity [m/s]	<0.01	<0.01	<0.01

**Table 3:** Results of the measurements performed with the condensation particle counter (CPC).

	Task 1	Task 2	Task 3	Background
Trial 1—Mean Particle number concentration [particles/cm^3^]	16,421.69	28,895.80	18,279.44	13,358.94
Trial 2—Mean Particle number concentration [particles/cm^3^]	16,716.12	37,568.52	22,708.98	

**Table 4:** Results of the application of CB Nanotool version 2.0 for the conditions of Trial 1.

**CB Factors**	**Task 1**	**Task 2**	**Task 3**
PM OEL	20 µg/m^3 1^	20 µg/m^3 1^	20 µg/m^3 1^
PM Carcinogenicity	No	No	No
PM Reproductive	Yes ^2^	Yes ^2^	Yes ^2^
PM Mutagenicity	No	No	No
PM Dermal Toxicity	Yes ^3^	Yes ^3^	Yes ^3^
PM Asthmagen	Yes ^4^	Yes ^4^	Yes ^4^
NM Surface Chemistry	unknown	unknown	unknown
NM Particle Shape	unknown	unknown	unknown
NM Particle Diameter	unknown	unknown	unknown
NM Solubility	unknown	unknown	unknown
NM Carcinogenicity	unknown	unknown	unknown
NM Reproductive Toxicity	unknown	unknown	unknown
NM Mutagenicity	unknown	unknown	unknown
NM Dermal Toxicity	unknown	unknown	unknown
NM Asthmagen	unknown	unknown	unknown
**Severity Score|Band**	**72|High**	**72|High**	**72|High**
Estimated amount of material used	>100 mg	>100 mg	>100 mg
Dustiness/mistiness	High	High	High
Number of employees with similar exposure	1–5	1–5	1–5
Frequency of operation	Daily	Daily	Daily
Duration of operation	<30 min	1–4 h	30–60 min
**Probability Score|Band**	**70|Likely**	**80|Probable**	**75|Likely**
**Overall Risk Level** **Without Controls**	**RL 3—Containment**	**RL 4—Seek** **Specialist Advice**	**RL 3—Containment**

^1^ Considering the lowest OEL recommended in Portugal: cobalt inorganic compounds [26]. ^2^ Repr. 2 (H361f) according to the material safety data sheet. ^3^ Skin Sens. 1 (H317) according to the material safety data sheet. ^4^ Resp. Sens. 1 (H334) according to the material safety data sheet.

**Table 5:** Results of the application of CB Nanotool version 2.0 for the conditions of Trial 2.

**CB Factors**	**Task 1**	**Task 2**	**Task 3**
PM OEL	200 µg/m^3 1^	200 µg/m^3 1^	200 µg/m^3 1^
PM Carcinogenicity	Yes ^2^	Yes ^2^	Yes ^2^
PM Reproductive	No	No	No
PM Mutagenicity	No	No	No
PM Dermal Toxicity	Yes ^3^	Yes ^3^	Yes ^3^
PM Asthmagen	No	No	No
NM Surface Chemistry	unknown	unknown	unknown
NM Particle Shape	unknown	unknown	unknown
NM Particle Diameter	unknown	unknown	unknown
NM Solubility	unknown	unknown	unknown
NM Carcinogenicity	unknown	unknown	unknown
NM Reproductive Toxicity	unknown	unknown	unknown
NM Mutagenicity	unknown	unknown	unknown
NM Dermal Toxicity	unknown	unknown	unknown
NM Asthmagen	unknown	unknown	unknown
**Severity Score|Band**	**63|High**	**63|High**	**63|High**
Estimated amount of material used	>100 mg	>100 mg	>100 mg
Dustiness/mistiness	High	High	High
Number of employees with similar exposure	1–5	1–5	1–5
Frequency of operation	Daily	Daily	Daily
Duration of operation	<30 min	1–4 h	30–60 min
**Probability Score|Band**	**70|Likely**	**80|Probable**	**75|Likely**
**Overall Risk Level** **Without Controls**	**RL 3—Containment**	**RL 4—Seek** **Specialist Advice**	**RL 3—Containment**

^1^ Considering the lowest OEL recommended in Portugal: nickel inorganic compounds [26]. ^2^ Carc. 2 (H351) according to the material safety data sheet. ^3^ Skin Sens. 1 (H317) according to the material safety data sheet.
